# A Comparison of Landraces vs. Modern Varieties of Lettuce in Organic Farming During the Winter in the Mediterranean Area: An Approach Considering the Viewpoints of Breeders, Consumers, and Farmers

**DOI:** 10.3389/fpls.2018.01491

**Published:** 2018-10-18

**Authors:** Joan Casals Missio, Ana Rivera, Maria Rosario Figàs, Cristina Casanova, Borja Camí, Salvador Soler, Joan Simó

**Affiliations:** ^1^Miquel Agustí Foundation, Campus del Baix Llobregat, Catalonia, Spain; ^2^Department of Agri-Food Engineering and Biotechnology, BarcelonaTech, Campus del Baix Llobregat, Catalonia, Spain; ^3^Instituto de Conservación y Mejora de la Agrodiversidad Valenciana, Universitat Politècnica de València, Valencia, Spain; ^4^Arrreu Tools for Agroecological Accompaniment, Barcelona, Spain

**Keywords:** agrobiodiversity, *Lactuca sativa* L., *Bremia lactucae*, participatory plant breeding, plant phenotyping

## Abstract

The interest of farmers in growing lettuce landraces is increasing, as landrace varieties prove particularly appealing to consumers striving to purchase natural, local, and high-quality produce. Although high genetic diversity exists in the landrace gene pool, this has scarcely been studied, thus hindering landrace utilization in agriculture. In this study, we analyzed the genetic diversity and the agronomic and quality traits of lettuce landraces in organic agrosystems, by characterizing 16 landraces and 16 modern varieties. We compared 29 morphological descriptors, and several traits relating to agronomic behavior (total and commercial weight, resistance to *Bremia lactucae*) and quality (color, chlorophyll, dry matter, and total sugars). Trials were conducted in two localities and managed following organic farming practices. Moreover, farmers and consumers participated in the phenotyping of accessions by scoring yield, resistance to *B. lactucae*, appearance, and taste acceptance. Results show that cultivar group, rather than the genetic origin (modern vs. landrace), is the major source of variation for all agronomic and quality traits. Batavia and Butterhead were highly homogeneous cultivar groups, while Cos accessions showed a much higher intra-varietal diversity. There was also a clear separation between modern and landrace varieties of Oak leaf. Fifteen out of the 16 evaluated landraces presented a high susceptibility to the particular *B. lactucae* race isolated from the experimental field - a new race not reported before. Breeding programs intended to introgress genetic resistance to this pathogen are a major priority to recover the cultivation of lettuce landraces. Principal component analysis (PCA), conducted on all quantitative data, showed a clear differentiation between modern varieties and landraces, mostly related to their commercial weight and susceptibility to *B. lactucae.* These seem the most important traits influencing farmer and consumer evaluations. Farmers showed a high capacity for characterizing the samples and agreed with consumers when scoring for the external appearance. It is proposed that farmers and consumers should be included in the phenotyping platforms in future research projects aiming for recovery of landraces.

## Introduction

Vegetable landraces (locally adapted, traditional plant varieties) have been generally displaced from market-driven production due to their lower yields, inferior pest and disease resistance, and poorer postharvest shelf life in comparison with modern varieties (van de Wouw et al., 2010). This has led to serious cultural and genetic erosion over the past 100 years (Negri, 2003; Hammer and Teklu, 2008). However, landraces are presently living a rebirth, driven by consumer demand for natural, local, and high-quality produce. New consumer groups, interested in purchasing quality foods linked to traditional and environmentally friendly labels, together with farmers concerned with the environmental and social impacts of food production, are rediscovering landraces as a source of value-added foods intrinsically associated with local production (Villa et al., 2005). Nevertheless, although significant efforts have been devoted in recent decades to collect and preserve landraces *ex situ* (Gepts, 2006), generally materials are stored in seed banks without any phenotypic information (Prada, 2009), thus hindering their utility to farmers. Therefore, it is of great importance to characterize these materials to make them available for commercial cultivation, and actualize their agronomic, sensory, and postharvest performances, to fit with current agriculture and consumption standards (Casañas et al., 2017). The classical approach for such characterization studies involves the phenotyping by research centers of the most important agronomic and quality traits, with the objective to describe yield performance and identify particular sensory or nutritional traits enhancing the distinctiveness of each variety. Nevertheless, to increase the worth of these studies to farmers, and include traits most relevant for consumers, the active integration of both of these groups in the phenotyping platform may offer a suitable alternative. This can be done through integration of sensory analysis (Tesfaye et al., 2013) and participatory plant breeding methodologies (Morris and Bellon, 2004) in a conjoint phenotyping platform with plant breeders.

The Iberian Peninsula is a hotspot for agrobiodiversity (Veteläinen and Maxted, 2009). Although for some crop species landraces are still present in the market [particularly for tomato (*Solanum lycopersicum* L.) and dry beans (*Phaseolus vulgaris* L.)], for other historically important crops, landraces are often enclosed in home gardens managed by old farmers (Casals et al., 2017). This is the case for lettuce (*Lactuca sativa* L.), an important leafy vegetable in European cuisine, which was domesticated in the eastern Mediterranean basin (Mou, 2008). Although it has great dietary and economic importance in Spain, the fourth greatest producing country in the world (Food and Agriculture Organization Corporate Statistical Database [FAOSTAT], 2016), and the richness of local cultivars have been preserved, landraces still remain marginal in the markets. In the area of study (Catalonia, NE Spain), several landraces were anciently appreciated, for instance, *cua d’oreneta* (“swallow tail”), *enciam del sucre* (“sugar lettuce”), *enciam negre* (“black lettuce”), or *enciam dels tres ulls* (“three eyed lettuce”). Most of these varieties remain cultivated in small areas, and others solely present in *ex situ* collections (Casals et al., 2017). To successfully recover the cultivation of lettuce landraces, there is a present need to investigate the genetic diversity at both phenotypic and molecular levels, which has been scarcely addressed in the scientific literature (Jansen et al., 2006; Vicente et al., 2008).

In contrast to other major crops, where significant increases in yield have been obtained by selecting for the harvested organ (seed, fruit, and tuber), higher lettuce biomass is not a trait generally present in the ideotypes of plant breeding programs (Still, 2007). For these species, the appearance of high-yielding modern varieties (i.e., producing a higher biomass per unit area of the harvested organ) seems not the principal factor driving the substitution of lettuce landraces, as has been the case for most other horticultural crops (van de Wouw et al., 2010). Other characteristics such as postharvest shelf life or resistance to pest and diseases have been more important in this process. Resistance to downy mildew (*Bremia lactucae* Regel) and lettuce aphid [*Nasonovia ribisnigri* (Mosley)] are currently the main characteristics driving lettuce breeding (Mou, 2008). Downy mildew is the most significant disease affecting lettuce, and the most efficient control strategy is the genetic resistance conferred by *Dm* genes (Michelmore and Wong, 2008). The gene-for-gene interaction between *L. sativa* and *B. lactucae*, and the pathogen variability, has led to continuous efforts of plant breeders to select for new resistance genes. So far, 28 *Dm* genes have been described, and modern lettuce varieties each carry a particular set of these genes (Parra et al., 2016). Usually farmers select the varieties to be cultivated based on the number of races for which one variety is resistant. Thus, the comparative lack of resistance to downy mildew in landraces (van Treuren et al., 2013) is the principal factor that has provoked their replacement by modern lettuce varieties. Other factors, such as cultivar diversification (some types are not present in the landrace gene pool), postharvest shelf life, and product standardization may also have had an important role.

Cultivation of lettuce is known to offer high profitability for farmers during the winter season (October-March) due to its resistance to cold temperatures, the minimal human labor needed during the crop cycle, and the lack of competence for agricultural land with other crops during this season. However, low temperatures and high humidity favor the incidence of downy mildew (Mou, 2008), making cultivation of non-resistant lettuce varieties during this season extremely difficult. Farmers interested in distinguishing their products in the food market are embracing organic farming and landrace labels, and desire landraces that show good agronomic and quality characteristics under these conditions. The objective of this study was to evaluate the genetic diversity and describe the agronomic performance and quality characteristics of lettuce landraces in organic agrosystems. We evaluated 16 landraces and 16 modern varieties of lettuce by means of a multi-stakeholder approach, including the participation of farmers (through participatory plant-breeding protocols) and consumers (through sensory analysis). This type of complex phenotyping platform enabled description of the principal differences between landraces and modern varieties, and identification of the key factors driving both farmer and consumer preferences. Moreover, the *B. lactucae* race present in the area was isolated, and the germplasm screened against isolates of this race.

## Materials and Methods

### Experimental Design

**FIGURE 1:**
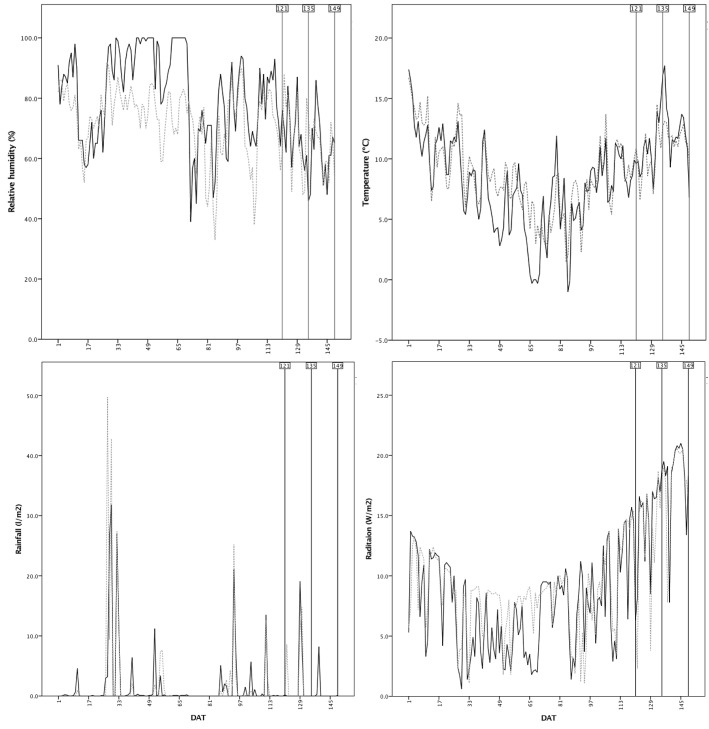
Climatic conditions registered in the assays (Benifallet: continuous line; La Múnia: dashed line) during the cropping season: mean daily values for relative humidity, temperature, rainfall, and radiation. Harvesting dates are indicated with vertical lines. DAT, days after transplant.

To represent the extensive germplasm available for organic farmers, seed companies, seed banks, and plant nurseries from the study area were interviewed. This resulted in the collection of a total of 32 genotypes belonging to different lettuce cultivar groups (Oak leaf, Butterhead, Batavia, and Cos) (**Table 1**). Landraces (16) and modern varieties (16) were represented equally in the study. Samples were grown during the winter season in two localities [Benifallet (40°58′22.46″N, 0°29′51.89″E) and La Múnia (41°19′26.8″N, 1°36′28.1″E)], separated by 120 km. These localities were selected to represent different agroclimatic conditions relevant to lettuce production in Catalonia (**Figure 1**). Trials were conducted in fields that had been managed following organic farming practices for at least 15 years. Previous farmer management of the field trials consisted in a crop rotation based in many botanical families, including Brassicaceae, Liliaceae, and in less proportion Chenopioideae and Asteraceae during the fall season, and Liliaceae, Cucurbitaceae, and Solanaceae during the spring season. More specifically, the rotation previous to the transplant was broccoli (*Brassica oleracea* L. var *italica*) – cucumber (*Cucumis sativus* L.) in Benifallet, and broccoli-aubergine (*Solanum melongena* L.) in La Múnia. Both localities have similar edaphic and irrigation water characteristics, with slightly basic soil and water, clay loam soils, and low organic matter content (2.3% Benifallet, 1.2% La Múnia), but differ in the content of several macronutrients (N, P, K, and Mg among others) (**Table 2**). Plants were irrigated with drip tapes (La Múnia) or sprinklers (Benifallet) and fertilized with a single application of cow manure prior to planting (equivalent of N 100 kg/ha). No phytosanitary treatments were applied during cultivation, and weeds were controlled manually. In each locality, a randomized block design was applied, with three replicates and 27 plants per plot, using a plant density of 6.67 plants/m^2^. The total crop cycle length was 149 days (transplantation: 26/10/2016, late harvest: 23/03/2017).

**Table 1:** List of accessions characterized.

ID^1^	Variety name	Accession^2^	Type	Donor	Cultivar group^3^	Earliness (DAT)^4^	Resistances^5^
13	Negre	FMA/113	LR	FMA	Batavia	130-135	
	Carxofet	FMA/112	LR	FMA	Batavia	112-122	
	Meravella	FMA/99	LR	FMA	Batavia	133-140	
	Meravella d’hivern		LR	Plant nursery (Pastoret)	Batavia	130-140	
9	Carxofet	FMA/5	LR	FMA	Butterhead	116-130	
11	De primavera	FMA/87	LR	FMA	Butterhead	123-135	
	Carxofet		LR	Plant nursery (Pastoret)	Butterhead	107-122	
	Negre	FMA/253	LR	FMA	Cos	124-134	
	D’hivern	FMA/252	LR	FMA	Cos	121-129	
	Del terreno	FMA/134	LR	FMA	Cos	135-140	
15	Negre de reus		LR	Plant nursery (Pastoret)	Cos	130-140	
16	Negre de Vilafranca		LR	Plant nursery (Pastoret)	Cos	114-122	
14	Negre borratger	386/935	LR	SIGMA	Cos	128-135	
10	Cua d’oreneta		LR	Plant nursery (Pastoret)	Oak leaf	113-130	
12	Francès	219/855	LR	SIGMA	Oak leaf	125-140	
	Fulla de roure	60/387	LR	SIGMA	Oak leaf	125-140	
2	Carmen		MV	Gautier	Batavia	133-140	LMV: 1
5	Magenta		MV	Gautier	Batavia	126-140	16, 21, 23, 32/LMV: 1
7	Novelsky		MV	Rijk Zwaan	Batavia	140-150	Bl: 16-28, 30-32, Nr: 0
	Arena		MV	Vilmorin	Batavia	130-140	
8	Pomery		MV	Gautier	Butterhead	114-122	Bl: 16-32/Nr: 0/LMV: 1
4	Janique		MV	Nunhems	Butterhead	117-126	Bl: 16-30, 32/Nr: 0
1	Abago		MV	Rijk Zwaan	Butterhead	115-122	Bl: 16-31/Nr: 0/LMV: 1
	Amboise		MV	Gautier	Lollo	128-140	Bl: 16-27, 29, 30, 32/Nr: 0
	Rivero		MV	Clause	Oak leaf	121-135	Bl: 1-28, 28, Nr: 0
	Camarde		MV	Gautier	Oak leaf	118-122	Bl: 16-32/Nr: 0/LMV: 1
	Kiari		MV	Nunhems	Oak leaf	130-145	Bl: 16-32/Nr: 0/Fol: 1 HR
	Navara		MV	Nunhems	Oak leaf	126-135	Bl: 16-26, 28, 32/Nr: 0
3	Conuai		MV	Rijk Zwaan	Oak leaf	121-135	Bl: 16-32/Nr: 0/LMV: 1
	Rutilai		MV	Rijk Zwaan	Oak leaf	115-122	Bl: 16-32/Nr: 0/LMV: 1
6	Mathix		MV	Vitalis	Oak leaf	115-122	Bl: 16-32/Nr: 0/Pb
	Horix		MV	Vitalis	Oak leaf	108-122	Bl: 16-29, 32/Nr: 0/Pb

*^1^Accessions evaluated by consumers. ^2^For genotypes obtained from seed banks (FMA, SIGMA), accession number is provided. ^3^Cultivar group according to UPOV classification (International Union for the Protection of New Varieties of Plants [UPOV], 2016). ^4^Earliness: number of days after transplant (DAT) (50% of plants reached the commercial stage) measured in La Múnia (first value) and Benifallet (second value). ^5^Genetic resistances according to the information provided by seed companies. LR, landrace; MV, modern variety.*

**Table 2:** Physical and chemical characteristics of soil and irrigation water in La Múnia and Benifallet field trials.

	Soil			Irrigation water		
	Benifallet	La Múnia	Units	Benifallet	La Múnia	Units
pH	8.2	8		7.5	8.4	
Electrical conductivity	0.367	0.336	dS/m	0.962	1.38	dS/m
Organic matter	2.34	1.24	%			
Ca	43.1	31.72	%CaCO_3_	7.26	4.89	meq/l
N-NO_3_	24	49	mg/kg	0.05	1.02	meq/l
P _(Olsen)_	33	151	mg/kg	4.61	<40	meq/l
K	428	205	mg/kg	0.05	<0.03	meq/l
Mg	252	378	mg/kg	3.58	7.5	meq/l
Ca	6422	4875	mg/kg	7.26	4.89	meq/l
Na	35	70	mg/kg	0.82	1.76	meq/l
Fe	2	0.56	mg/kg	<1	<25	meq/l
Mn	1.5	2.21	mg/kg	0.13	<0.1	meq/l
Textural class	Clay loam	Clay loam				

### Morphological Descriptors

Accessions were visually classified in the different cultivar groups using the classification proposed by the International Union for the Protection of New Varieties and Plants (International Union for the Protection of New Varieties of Plants [UPOV], 2016; **Table 1**). A total of 29 morphological descriptors were recorded for each accession, assessing different parts of the plant: cotyledons (color, anthocyanin presence, and shape), young leaf (position, color, anthocyanin distribution and intensity of coloration, blade border, and shape (outline, apex, base, and margin), vertical margin, undulation, and venation), adult outer leaf (color, anthocyanin distribution, and intensity, glossiness on the upper side, surface profile, blade border and shape (outline, apex, base, and margin), depth of incisions, blistering), head (head formation, shape in vertical section, overlapping of leaves), flower, and inflorescence, as proposed by Kristkova et al. (2008).

### Agronomic Characterization

For each accession and locality, earliness was visually evaluated, and measured as the number of days between the transplant and the moment when 50% of plants reached the commercial stage [expressed as the number of days after transplant (DAT)]. According to these results, early-, mid-, and late-harvests were conducted at 121, 135, and 149 days after transplantation, in order to measure yield related traits during the length of the harvesting period, following the standard practices of farmers. In each harvest, 12 randomly selected individuals per accession (four from each block) were weighed. Both total weight (in g) and commercial weight (i.e., after external, old, and damaged leaves were removed according to typical farming practices; % of the total weight) were obtained. Incidence of *B. lactucae* was assessed on a per-plant basis at each harvest date using the following scale: 0 (no symptoms), 1 (few, small lesions), 2 (less than half of leaves with lesions), and 3 (high incidence, sporulating profusely), as proposed previously (Gustafsson, 1989).

### Color and Chemical Evaluation

At mid-harvest, from the locality of La Múnia, three lettuces per accession were sampled and immediately processed for chemical and color analyses. Color (expressed as *L*^∗^ (luminosity), *a*^∗^ (ranging from green [negative values] to red [positive values]), *b*^∗^ (ranging from blue [negative values] to yellow [positive values]) coordinates of the CIELAB color space), and chlorophyll content [measured as the index of absorbance difference (*I*_AD_)] of each accession were measured in the equatorial and terminal parts of three randomly selected inner leaves. A Konica Minolta CR-410 (Minolta, Osaka, Japan) and a DA-meter (TR-Turoni, Forli, Italy) were used for these analyses, respectively, with means of the three measurements used as the definitive result.

For chemical analyses, outer old leaves and the lettuce core were removed, with the remaining leaves washed in cold, running tap water. These samples were cut into pieces of approx. 2 cm^2^ using a sharp stainless steel knife. Dry matter content was measured by drying the samples in an air oven (65°C, 72 h) and then weighing. For sugar analysis, 50 g of cut lettuce sample and 15 g of deionized water were mixed and homogenized in a blender. The addition of water was necessary to achieve a homogeneous sample. Sugars were extracted using deionized water. Approx. 30 g of homogenate was mixed with 20-30 mL of water, shaken for 15 min, and centrifuged. This was repeated three consecutive times and the three filtrated supernatants collated to give a volume of 100 mL of extract. Glucose, fructose, and sucrose were analyzed by HPLC, equipped with a pump (Beckman 110B, San Ramon, CA, United States), an injector (Hewlett Packard Serie 1100, Walbrom, Germany) and a Refractive Index Detector (Beckman 156, United States). A Luna NH2 column, 250 mm × 4.6 mm (Phenomenex, Torrance, CA, United States) was used. Results are expressed as total sugars [mg/g fresh weight (fw)].

### Screening for Resistance to *Bremia lactucae*

A lettuce from the La Múnia field showing a high incidence of *B. lactucae* (sporulating profusely) was harvested and brought to the laboratory. Conidiophores were extracted from the affected plant, and the isolate reproduced in the susceptible Green Towers variety. Once abundant new sporulations had been reproduced in these plants, these were used for characterization of the Catalonian *B. lactucae* isolate. Fifteen differential genotypes, defined by the International Bremia Evaluation Board (IBEB,^1^ verified 25 June 2018), were used to help characterize the isolate. Inoculum was prepared for plant screening by shaking cotyledons bearing 3- to 4-day-old conidiophores with conidia in sterile distilled water. Seeds of screened lettuce varieties were sown in 40 cm × 30 cm × 10 cm trays filled with saturated substrate (30% white peat, 70% black peat; Neuhaus Huminsubstrat N3, Lassmann-Dellmann). Seedlings with fully expanded cotyledons (approx. 9-10 days after sowing) were inoculated by a sprayer with a suspension of 2 × 10^5^ conidia/mL. Twenty plantlets of each variety were inoculated in three replicated experiments. After inoculation, the trays were covered with transparent plastic bags to create 100% humidity. Incubation was performed in a growth chamber under standard conditions, with a light intensity of 4000-5000 lux, continuous temperature of 16°C, and a 12-h photoperiod. The seedlings were observed at 7, 10, and 15 days after inoculation. Each plant variety was then scored for necrosis or asexual sporulation produced by *B. lactucae*. In the case of sporulation, four levels were established: 0 (absence of sporulation), 1 (weak sporulation, sporulation less than susceptible control), 2 (sparse sporulation), and 3 (sporulation comparable to the susceptible control). An accession was considered positive (exhibiting susceptibility to infection) when at least 5% of the tested plants gave a level of sporulation more than 2.

Finally, by using the same methodology as described above, we screened the experimental germplasm (**Table 1**) against the *B. lactucae* previously isolated. With this aim 160 plants per accession, divided in six replicates, were inoculated and the susceptibility to *B. lactucae* assessed. Results are expressed as the % of susceptible plants in each accession. In these experiments, we included Olaf variety as a susceptible control.

### Farmer and Consumer Evaluations

With the aim of incorporating farmers in the characterization of the accessions, a farmer evaluation was organized in the field of La Múnia with the participation of 22 farmers. Participants evaluated visually the experimental plots, without any information regarding the name of the variety nor the origin (blind evaluation) and scored the accessions for the traits “commercial value” in a scale ranging from 0 (not interesting accession) to 10 (highly interesting accession) and “resistance to *B. lactucae*” in a scale ranging from 0 (non-resistant accession, high incidence) to 10 (resistant accession, without symptoms). In parallel, a consumer survey (untrained panellists) was organized in the sensory laboratory of the Barcelona School of Agricultural Engineering, with the participation of 47 consumers (55% female, 45% male; 45% between 19 and 34 years, 35% between 35 and 55 years, 20% between 56 and 70 years). Solely regular consumers of lettuce (at least one time per week) were selected, regardless of whether they were regular consumers of organic products (15% of participants). Each panelist received a whole lettuce to evaluate appearance and a cut sample to evaluate taste. Out of the 32 accessions, 16 (eight landraces and eight modern varieties) were rated on a 10 cm semi-structured scale from 0 (“Dislike”) to 10 (“Extremely like”) for “external appearance” and “taste acceptance” traits. Accessions were distributed randomly in two tasting sessions, in each of which half of the materials were evaluated. Samples were coded with a random three-digit number. Panellists did not receive any information regarding the objective of the study, neither about the origin of the varieties. Tasting sessions were carried out in a room designed for sensory analyses (International Organization for Standardization [ISO], 2017), using white light for the “external appearance” test and green light to mask the color during the “taste acceptance” test.

### Statistical Analyses

Yield data (total weight and commercial weight) was analyzed within each locality and at each harvesting date by means of analysis of variance (ANOVA), using a full factorial model. We performed two independent ANOVA with the objective to assess (i) differences between cultivar groups [factors: accession (cultivar group), cultivar group, and block] and (ii) differences between origins (landrace or modern variety) within each cultivar group [factors: accession (origin), origin, and block]. Harvesting date and locality factors were not considered in the model, in order to obtain a more detailed description of the agronomic behavior of the accessions in each locality.

Resistance to *B. lactucae*, both in laboratory and field tests, and evaluations performed by farmers and consumers were analyzed by means of ANOVA considering solely the accession factor. For farmer and consumer data, each individual score was considerate as a replicate for the analysis. For significant factors, mean separation was conducted using the Student-Newman-Keuls test (*snk*, *p* < 0.05). A hierarchical cluster analysis (HCA), with average linkage applied as the grouping method, was carried out using Pearson distances for quantitative traits (chlorophyll, color, total sugar content, and dry matter) and Jaccard’s distances for qualitative variables (morphological descriptors). Results were presented using a dendrogram by means of the same software. Principal component analysis (PCA) and Pearson bivariate correlation analyses were used to assess the variables underlying consumer and farmers preferences. SPSS (v.12.0, SPSS Inc., Chicago, IL, United States), Acuity (v.4.9, Axon Instruments, Union City, CA, United States), and R (R core team 2017; Agricolae, PCAmethods, and Ellipse packages) statistical programs were used for univariate (ANOVA, mean separation), cluster, and PCA analyses, respectively.

## Results

### Classification of Landraces According to Morphological Descriptors

Out of the 32 accessions studied, 11 belonged to Oak leaf, seven to Batavia and Cos, six to Butterhead, and one to Lollo cultivar groups (**Table 1**). The Amboise cultivar was initially included in the assay due to its classification in the Batavia group (according to the seed company description), but it was further reclassified as a Lollo cultivar. In each cultivar group both modern varieties and landraces were represented, except in the case of the Cos group, were solely landraces were identified. This was due to the particular interest of organic farmers in the *enciam negre* (“black lettuce”) landrace, and the lack of available organic seeds of commercial cultivars in this group. The traditional names of landraces were highly diverse and did not offer appropriate information regarding the cultivar group pertinence. Such names referred to the crop cycle [e.g., *D’hivern FMA/252* (“winter lettuce,” Cos); *De primavera FMA/87* (“spring lettuce,” Butterhead)], origin [e.g., *Francès SIG/219/855* (“french lettuce,” Oak leaf)] or specific morphological traits such as leaf type [e.g., *Cua d’oreneta* (“swallow tail,” Oak leaf)], and color [e.g., *Enciam negre* (“black lettuce,” five accessions, all belonging to the Cos type)]. In some cases, the same traditional name corresponded to multiple distinct cultivar groups, for instance, the *Carxofet* (“little artichoke”) accessions, two of which were classified as Batavia and one as Butterhead.

**FIGURE 2:**
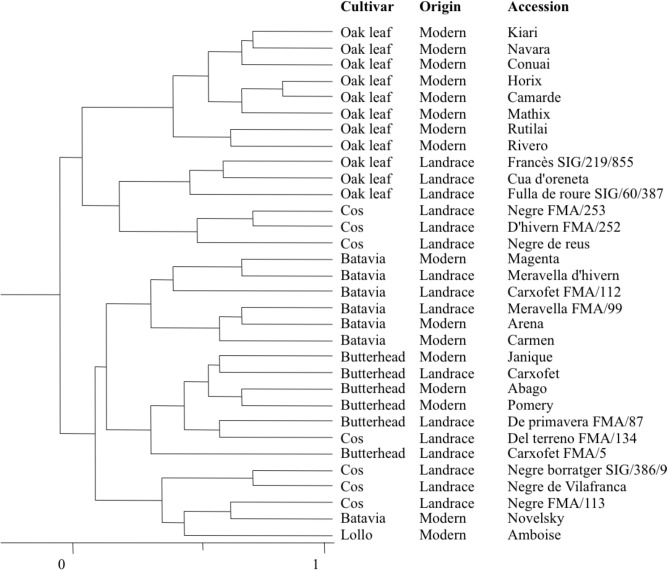
Relativeness between accessions according to the hierarchical cluster analysis performed with 29 morphological descriptors.

The groups obtained by means of HCA on the 29 morphological descriptors studied (**Figure 2**) were highly consistent with the cultivar group pertinence in the case of Batavia, Butterhead, and Oak leaf. Batavia and Butterhead were the most homogeneous cultivar groups, with all of the accessions belonging to each group clustering together in the HCA [with the exception of Novelsky - this Batavia type was more related to Amboise (Lollo) and *Negre FMA/113* (“black lettuce,” Cos)]. Within the Oak leaf group, two clusters were identified, clearly separating landraces from modern varieties. Cos seemed a highly divergent group, forming two distinct clusters [one more related to Oak leaf landraces, and the other to Amboise (Lollo)]. Finally, one Cos accession [*Del terreno FMA/134* (“field lettuce”)] clustered together with the Butterhead group.

### Agronomic Characterization

Earliness ranged from 107 to 140 DAT in La Múnia and from 122 to 150 DAT in Benifallet, with Butterhead cultivar group showing the highest earliness in both localities (significantly different to the other groups at *p* < 0.05, except with Oak leaf) (**Table 1**). According to these results we decided to perform 3 harvests (early-harvest, 121 DAT; mid-harvest, 135 DAT; late-harvest: 149 DAT) with the objective to assess the yield of each accession during the length of the harvesting period. Results for total weight (g) and commercial weight (%) revealed that major differences were related to cultivar groups rather than to the genetic origin (landrace vs. modern) of the accessions (**Table 3** and **Supplementary Table S1**). In both localities, and regardless of the harvesting date, the higher values for total weight were obtained by Cos and Butterhead accessions. No general pattern for the modern/landrace comparison was found. For example, landraces yielded significantly higher total weights in the Oak leaf cultivar group, while in the case of Butterhead, agronomic behavior was highly dependent on the locality (with higher landrace yields in Benifallet, and lower in La Múnia), signaling an important G × E effect. No significant differences were found between landraces and modern varieties in the Batavia group.

**Table 3:** Comparisons between cultivar groups, and between genetic origins (landraces vs. modern varieties) within cultivar groups, for the agronomic traits studied [total weight (g) and commercial weight (%)] in *Lactuca sativa* L. accessions grown in (a) La Múnia, and (b) Benifallet.

	Early-harvest				Mid-harvest				Late-harvest			
	Total weight (g)		Commercial weight (%)		Total weight (g)		Commercial weight (%)		Total weight (g)		Commercial weight (%)	
**(a) La Múnia**											
**Cultivar groups**											
Batavia	299.5	c	78.5	b	398.4	c	77.6	ns	617.4	c	86.2	a
Butterhead	359.6	b	81.7	b	480.8	b	78.8	ns	640.3	b	82.8	b
Cos	477.8	a	78.5	b	567.0	a	75.9	ns	783.8	a	81.3	b
Oak leaf	244.8	d	84.4	a	332.9	d	81.6	ns	441.8	d	83.0	b
**Origin**											
Batavia											
Modern	304.2	*ns*	79.3	*ns*	397.5	*ns*	78.4	*ns*	592.8	ns	85.1	ns
Landrace	293.5		77.8		399.9		76.5		649.4		87.7	
Butterhead											
Modern	327.6	^∗^	84.9	^∗∗^	452.0	*ns*	81.1	*ns*	602.6	^∗^	85.8	^∗^
Landrace	391.6		78.5		509.6		76.5		678.0		79.8	
Oak leaf											
Modern	203.2	^∗∗∗^	85.7	ns	280.0	^∗∗∗^	83.8	^∗^	371.3	^∗∗∗^	82.8	*ns*
Landrace	358.8		80.9		473.9		76.9		619.3		83.5	
**(b) Benifallet**											
**Cultivar groups**											
Batavia	180.8	b	79.2	a	445.8	b	89.2	a	578.1	c	92.2	a
Butterhead	369.6	a	82.2	a	779.9	a	79.4	c	742.6	b	78.9	c
Cos	376.7	a	73.7	b	789.3	a	77.5	c	893.6	a	78.2	c
Oak leaf	193.3	b	78.3	a	512.1	b	81.6	b	537.2	c	85.5	b
**Origin**											
Batavia											
Modern	188.5	*ns*	77.9	*ns*	456.9	*ns*	89.4	*ns*	571.1	*ns*	92.0	ns
*Landrace*	170.6		80.9		431.2		88.9		588.4		92.5	
Butterhead											
*Modern*	417.8	*^∗∗∗^*	92.2	^∗∗^	790.8	*^∗∗∗^*	81.9	^∗∗^	935.4	^∗^	81.9	^∗^
*Landrace*	321.4		72.4		680.7		76.5		604.9		76.5	
Oak leaf											
*Modern*	165.9	*^∗∗∗^*	80.2	*^∗∗∗^*	437.5	*^∗∗∗^*	84.0	*^∗∗∗^*	410.7	*^∗∗∗^*	86.4	*ns*
*Landrace*	266.2		73.5		711.0		75.3		874.4		83.2	

*Data were collected at early, mid, and late-harvests (121, 135, and 149 days, respectively). Within columns, letters indicate significant differences between corresponding cultivar groups (Student-Newman-Keuls test, at p < 0.05), and ^∗∗∗^p < 0.001, ^∗∗^p < 0.01, ^∗^p < 0.05, and ns (not significant) indicate significant differences between landrace and modern genotypes within each cultivar group.*

Commercial weight (%) was more dependent on the harvesting date and also showed a clearer separation between traditional and modern varieties. In all of the cases studied where significant differences were detected, higher commercial weights were recorded in the modern accessions. However, it should be noted that commercial weight was higher than 70% (i.e., 30% of the total weight should be discarded prior to commercialization) for all accessions, and even for accessions with severe reduction of the total weight [e.g., Arena (69.8%) or *Negre borratger SIG/386/935* “black lettuce” (72.4%)], harvested lettuces reached the minimum standards for commercialization.

### Chemical and Color Evaluation

**FIGURE 3:**
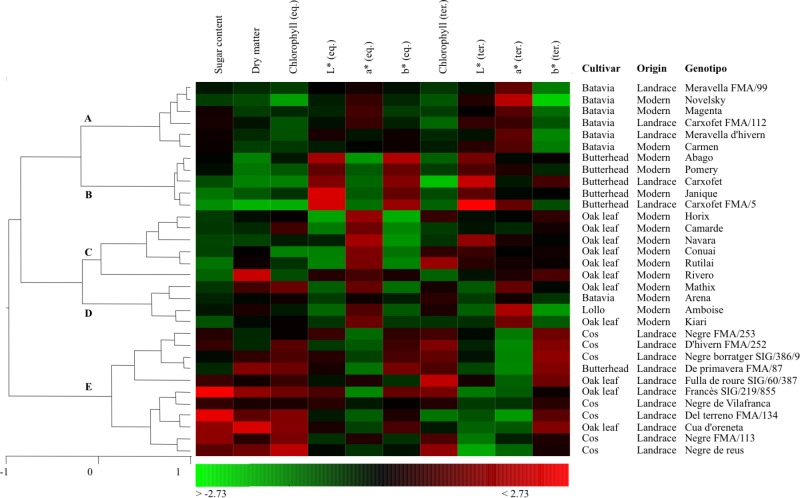
Hierarchical cluster analysis from chemical and color traits. Values are represented as a heatmap according to the scale below. eq., measured in the equatorial part of the leaf; ter., measured in the terminal part of the leaf.

Analogously with the results from the morphological characterization (**Figure 2**), HCA performed on color and chemical composition revealed a consistent clustering of the cultivar groups (**Figure 3**). The principal factor of classification (groups A-D vs. group E) was found to be related to the chemical composition (sugar content, dry matter, and chlorophyll) and to intensity of red color (*a*^∗^ coordinate) measured in the terminal part of the leaf. Cos and Oak leaf landraces, and one traditional Butterhead accession, clustered together (group E), and were characterized by high levels of sugars, dry matter, chlorophyll content and yellow color (*b*^∗^ coordinate, measured in the terminal part of the leaf), and low levels of red color (*a*^∗^ coordinate, terminal). Batavia (group A) and Butterhead (group B) accessions showed some relativeness in comparison with the rest of the collection, being characterized by low levels of sugars, dry matter, and chlorophyll content. Nevertheless, the two cultivar groups were distinct with respect to their color traits: luminosity (*L*^∗^ coordinate, both equatorial, and terminal) and yellow color (*b*^∗^ coordinate, equatorial) were higher in Butterhead accessions, while red color (*a*^∗^ coordinate, terminal) was higher in Batavia accessions. Most of the Oak leaf modern varieties clustered together (group C), characterized by their color profile in the equatorial part of the leaf [high values for red color (*a*^∗^) and low values for luminosity (*L*^∗^) and yellow (*b*^∗^)], but with a similar chemical profile to Butterhead and Batavia groups. Thus, a clear separation between Oak leaf modern varieties and landraces was observed, with landraces characterized by higher sugar, dry matter, and chlorophyll content, and modern varieties showing a more intense red color (*a*^∗^) in the equatorial and terminal part of the leaves. Finally, a more heterogeneous group (group D), formed by modern varieties of Oak leaf (Mathix, Kiari), Batavia (Arena), and Lollo (Amboise) cultivar groups, showed a similar profile to the Oak leaf group (group C), but with some differences related to the color at the terminal part of the leaves.

### Resistance to *Bremia lactucae*

The Catalonian isolate of *B. lactucae* showed no correspondence to any of the races previously reported by the IBEB, indicating that a previously undescribed race is present in the fields of this area. Regarding the susceptibility of the experimental germplasm, significant differences between accessions were detected, both in tests performed in the laboratory and in the field. All of the landraces, except *De primavera FMA/87* (“spring lettuce,” Butterhead type), were susceptible to the *B. lactucae* race isolated from the area (**Table 4**). Moreover, five of the 16 modern varieties studied were also susceptible. Results obtained in the laboratory were significantly correlated (*p* < 0.001) with field observations carried out by researchers in both localities [Benifallet (*r* = 0.62) and La Múnia (*r* = 0.79)] (**Table 5**), although in some cases, slightly different responses between laboratory and field tests were identified. In most of such cases, accessions characterized as susceptible in the laboratory were classified as intermediately resistant in the field. The higher correlation between the laboratory and La Múnia tests is consistent with the fact that the *B. lactucae* race used in the laboratory test was isolated from this particular field.

**Table 4:** Susceptibility of *Lactuca sativa* L. accessions to the *Bremia lactucae* pathogen, as scored in laboratory and field studies.

				Laboratory test		Field test (0–3)				
Variety	Accession	Origin	Cultivar group	Susceptible plants (%)		Benifallet		LaMunia		Resistance (qualitative)
1	Conuai	Modern	Oak leaf	0	f	0.1	j	0.5	hij	R-R-R
2	Rutilai	Modern	Oak leaf	0	f	0.0	j	0.5	hij	R-R-R
3	Abago	Modern	Butterhead	0	f	0.1	j	0.2	j	R-R-R
4	Novelsky	Modern	Batavia	0	f	1.3	gh	1.5	abcdefg	R-IR-IR
8	Pomery	Modern	Butterhead	0	f	0.1	j	0.2	j	R-R-R
9	Camarde	Modern	Oak leaf	0	f	0.0	j	0.5	hij	R-R-R
10	Amboise	Modern	Lollo	0	f	0.2	j	0.2	j	R-R-R
13	Janique	Modern	Butterhead	0	f	0.4	ij	0.3	j	R-R-R
15	Mathix	Modern	Oak leaf	0	f	1.7	fg	0.4	ij	R-S-R
24	De primavera FMA/87	Landrace	Butterhead	0	f	2.0	def	0.8	ghij	R-S-IR
16	Horix	Modern	Oak leaf	6	f	0.1	j	0.2	j	R-R-R
12	Kiari	Modern	Oak leaf	16	f	0.1	j	0.4	ij	R-R-R
23	D’hivern FMA/252	Landrace	Cos	39	e	2.6	abc	1.5	abcdefg	S-S-S
31	Carxofet	Landrace	Butterhead	47	de	2.6	abcd	1.2	cdefgh	S-S-IR
7	Carmen	Modern	Batavia	52	cde	0.7	hij	1.2	cdefg	S-IR-IR
18	Negre borratger SIG/386/935	Landrace	Cos	65	bcd	2.9	a	2.1	a	S-S-S
30	Negre de Vilafranca	Landrace	Cos	65	bcd	2.8	ab	2.1	ab	S-S-S
19	Francès SIG/219/855	Landrace	Oak leaf	67	bcd	2.0	cdef	1.6	abcdef	S-S-S
20	Negre FMA/113	Landrace	Cos	67	bcd	2.8	ab	1.8	abcd	S-S-S
22	Carxofet FMA/112	Landrace	Batavia	67	bcd	0.9	hi	1.1	defgh	S-IR-IR
29	Negre de reus	Landrace	Cos	67	bcd	2.5	abcde	1.5	abcdefg	S-S-S
17	Fulla de roure SIG/60/387	Landrace	Oak leaf	70	bcd	2.6	abc	1.7	abcde	S-S-S
28	Meravella d’hivern	Landrace	Batavia	72	bcd	1.0	hi	1.3	cdefg	S-IR-IR
21	Negre FMA/253	Landrace	Cos	73	bcd	2.9	a	1.8	abcd	S-S-S
11	Magenta	Modern	Batavia	77	abc	1.2	gh	1.2	cdefgh	S-IR-IR
25	Meravella FMA/99	Landrace	Batavia	77	abc	0.7	hij	1.3	bcdefg	S-IR-IR
32	Cua d’oreneta	Landrace	Oak leaf	79	abc	1.9	ef	1.0	efghi	S-S-IR
14	Navara	Modern	Oak leaf	82	ab	2.7	abc	1.2	cdefgh	S-S-IR
6	Arena	Modern	Batavia	89	ab	0.7	hij	1.6	abcdef	S-IR-S
5	Rivero	Modern	Oak leaf	89	ab	2.8	ab	1.9	abc	S-S-S
Olaf	Olaf	Control		100	a					S–
26	Del terreno FMA/134	Landrace	Cos			2.2	bcdef	0.9	fghij	-S-IR
27	Carxofet FMA/5	Landrace	Butterhead			2.6	abc	1.4	abcdefg	-S-IR

*The table includes variety identification number, accession name, origin (modern or landrace), cultivar group, and susceptibility to infection in laboratory testing (% of susceptible plants, six replicates), and in Benifallet and La Múnia field studies (graded 0-3, 26 replicates). Field study grading was carried out as follows: 0 (no symptoms), 1 (few, small lesions), 2 (less than half of leaves with lesions), and 3 (high incidence, sporulating profusely). Final laboratory-Benifallet-La Múnia susceptibility scores are reported as R, resistant; IR, intermediate resistance; S, susceptible. Within columns, letters indicate significant differences between accessions (Student-Newman-Keuls test, at p < 0.05).*

**Table 5:** Pearson bivariate correlations between agronomic, chemical, and color traits, together with farmer and consumer evaluations.

	Commercial weight (%)	Susceptibility to B. lactucae (Laboratory test, %)	Susceptibility to B. lactucae (Benifallet field, %)	Susceptibility to B. lactucae (La Mnia field, %)	Total sugars (mg/ g fw)	Dry Matter (%)	Commercial value (farmer)	Resistance (farmer)	Appearance (consumer)	Taste acceptance (consumer)	Chlorophyll (eq.)	L^∗^ (eq.)	a^∗^ (eq.)	b^∗^ (eq.)	Chlorophyll (ter.)	L^∗^ (ter.)	a^∗^ (ter.)	b^∗^ (ter.)
Total weight (g)		0.^∗^	0.62^∗∗∗^	0.51^∗∗^	0.57^∗∗∗^			−0.6^∗∗∗^	−0.5^∗^			0.43 ^∗^	−0.66^∗∗∗^	0.51^∗∗^			−0.51^∗∗^	
Commercial weight (%)		−0.49^∗∗^	−0.53^∗∗^	−0.70^∗∗∗^				0.49^∗∗^										
Susceptibility to *B. lactucae* (Laboratory test, %)			0.62^∗∗∗^	0.79^∗∗∗^	0.42^∗^			−0.70^∗∗∗^										
Susceptibility to *B. lactucae* (Benifallet field, %)				0.78^∗∗∗^	0.35^∗^			−0.83^∗∗∗^					−0.35^∗^				−0.5^∗∗^	0.53^∗∗^
Susceptibility to *B. lactucae* (La Múnia field, %)								−0.81^∗∗∗^										
Total sugars (mg/g fw)						0.49^∗∗^		−0.37^∗^			0.65^∗∗∗^		−0.38^∗^			−0.60^∗∗∗^	−0.52^∗∗^	
Dry matter (%)											0.65^∗∗∗^				0.40^∗^	−0.77^∗∗∗^	−0.41^∗^	0.45^∗^
Commercial value (farmer)								0.50^∗∗^	0.56^∗^									
Resistance (farmer)																		
Appearance (consumer)													0.59^∗^	−0.60^∗^				
Taste acceptance (consumer)																		
Chlorophyll (eq.)															0.55^∗∗^	−0.77^∗∗∗^	−0.67^∗∗∗^	0.57^∗∗∗^
*c*^∗^ (eq.)													−0.78^∗∗∗^	0.87^∗∗∗^	−0.39^∗^	0.46^∗∗^		
*a*^∗^ (eq.)														−0.94^∗∗∗^			0.49^∗∗^	
*b*^∗^ (eq.)																		
Chlorophyll (ter.)																−0.52^∗∗^	−0.44^∗^	0.38^∗^
*c*^∗^ (ter.)																		
*a*^∗^ (ter.)																		−0.90^∗∗∗^
*b*^∗^ (ter.)																		

*Solely significant correlations are shown. ^∗∗∗^, ^∗∗^, and ^∗^ indicate levels of significance as p < 0.001, p < 0.01, and p < 0.05, respectively.*

### Farmer and Consumer Preferences

An ANOVA performed on farmer and consumer evaluations revealed significant differences between accessions for all of the traits under study (*p* < 0.05), and differences between origins (landraces vs. modern varieties) for the traits “resistance to *B. lactucae*” (field evaluation made by farmers; modern varieties yielding higher scores) and “external appearance” (laboratory evaluation made by consumers; modern varieties being higher scored).

**FIGURE 4:**
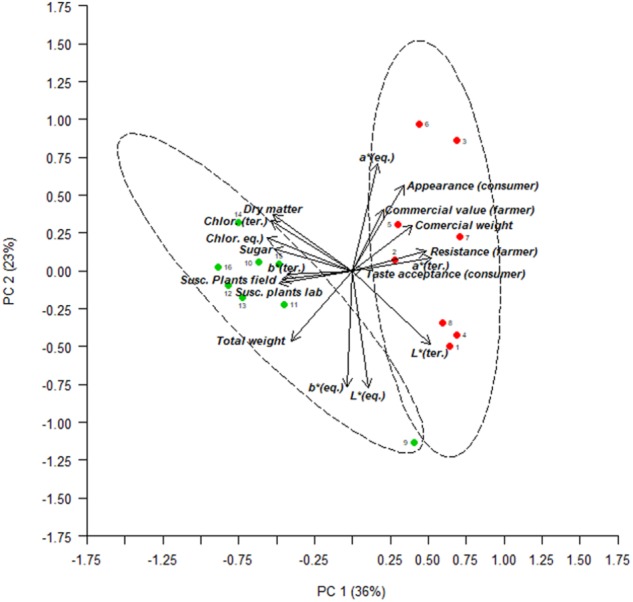
Plot from the two first principal components (59% of the total variation) in the PCA estimated from all the data of the experiment (agronomic, chemical, color, and resistance to *B. lactucae*, plus consumer and farmer evaluations), considering the 16 accessions evaluated by consumers (codes are presented in **Table 1**). Green points, landraces; red points, modern varieties.

To introduce farmer and consumer evaluations, a multivariate analysis was conducted with all of the data from the experiment recorded in the field of La Múnia (except for morphological descriptors). The first two components of the PCA, which accounted for 59% of the total variation, were plotted (**Figure 4**). PC1 (36% of the total variation), which was positively correlated to commercial weight, and negatively to susceptibility to *B. lactucae* (both for laboratory and field tests), provided a clear distinction between landraces and modern varieties. Moreover, farmers’ evaluations regarding the commercial value and resistance to *B. lactucae* variables, and consumers’ ratings (regarding external appearance) showed a clear tendency to prefer modern varieties, being sensitive to plants with intact leaves and negatively influenced by total weight trait. Some varieties such as Mathix (Oak leaf), Conuai (Oak leaf), or Novelsky (Batavia) seem to fit with farmer and consumer preferences. Consumer evaluations on taste acceptance were not discriminant between accessions nor origins, according to the PCA analysis.

For a greater understanding of the phenotypic traits underlying farmer and consumer preferences, a Pearson correlation analysis was conducted, considering all of the traits evaluated (**Table 5**). Farmer evaluations regarding the resistance to *B. lactucae* were highly correlated to the susceptibility tests performed in the laboratory (*r* = −0.70) and in the field (La Múnia *r* = −0.83; Benifallet *r* = −0.81), signaling their strong ability to discriminate between accessions regarding this trait. The sign of the correlation (negative) is due to the different scales of evaluation used by researchers (susceptibility) and farmers (resistance). Resistance measured by farmers was also correlated with total weight (*r* = −0.60), commercial weight (*r* = 0.49), and with their perception of the commercial value of each accession (*r* = 0.50), signaling that this group of characteristics drive farmers’ preferences for lettuce cultivars. With regard to consumer evaluations, few significant correlations were obtained. External appearance correlated positively with commercial value scored by farmers (*r* = 0.56) and with red color (*a*^∗^, equatorial, *r* = 0.59), and negatively with yellow color (*b*^∗^, equatorial, *r* = −0.60).

Regarding relationships between agronomic, chemical, and color traits, most of the correlations were detected between the color coordinates *L*^∗^, *a*^∗^, and *b*^∗^ (equatorial/terminal). Total sugars were correlated with chlorophyll content measured at the equatorial part of the leaf (*r* = 0.65), but not when measured at the terminal part. Moreover, sugars were also related to the color of the leaves, showing significant and negative correlations with red color (*a*^∗^) measured at the equatorial (*r* = −0.38) and terminal (*r* = −0.52) positions, and with luminosity (*L*^∗^) at the terminal position only (*r* = −0.60). Finally, chlorophyll content (equatorial and terminal) was also related to *L*^∗^, *a*^∗^, and *b*^∗^ color coordinates, when evaluated in the terminal part of the leaf.

## Discussion

In comparison with other major horticultural crops, genetic and phenotypic profiles of lettuce landraces have been scarcely studied in the scientific literature. Landrace varieties of crops are rapidly regaining importance in the commercial field, promoted mainly by the interest of specific niche markets, such as organic food production. Organic farmers are therefore interested in identifying lettuce landrace varieties (i.e., pure lines) that show promising agronomic performance, while also presenting distinctive organoleptic and nutritional quality traits. Our study shows that, when comparing landraces with modern varieties, the major source of variation is the cultivar group rather than the origin of the material. Moreover, in our study, we characterized the *B. lactucae* race present in the experimental field of La Múnia, which showed no correspondence with any of the previously reported races (Parra et al., 2016). Landraces were highly susceptible to this race, both when assessed in the field and in the laboratory (using inoculated plants), as solely one of the 16 landraces evaluated [*De primavera FMA/87* (“spring lettuce,” Butterhead type)] showed resistance to this pathogen. This accession can be considered a “traditionalized” modern variety (i.e., a modern variety that has been multiplied by farmers and recently adopted as a traditional variety), although this remains unclear. By contrast, most of the modern varieties showed good levels of resistance (with only five out of 16 exhibiting susceptibility), signaling that the genetic resistance conferred by *Dm* genes, already introgressed in modern cultivars, is functional against this new race. Susceptibility to *B. lactucae* is the major drawback currently limiting the cultivation of landraces by farmers (van Treuren et al., 2013). Therefore, breeding programs directed at introducing genetic resistance to landraces is a priority with the objective of recovering the cultivation of these varieties. Prior to undertaking these breeding programs, the composition and distribution of Catalonian *B. lactucae* isolates should be analyzed, and later decide which genes should be strategically introduced into the improved landraces. Nevertheless, despite the higher incidence of *B. lactucae* in landraces, all of the landraces studied reached commercially acceptable standards in this experiment. For some landraces, commercial weight reached only 70% of the total weight, but this was compensated by a higher total biomass production.

With respect to the quality traits compared in this study (total sugars, dry matter, and chlorophyll content), the higher scores were identified in landraces. Some accessions such as *Francès SIG/219/855* (“french lettuce,” total sugars: 15.6 mg/g fw) or *Del terreno FMA/134* (“field lettuce,” 15.2 mg/g fw), among others, showed promising values regarding sugar content when compared with the remaining accessions of the experiment (range of variation: 5.2-12.9 mg/g fw) and with results obtained by other authors (Still, 2007; Ouzounidou et al., 2013; López et al., 2014). Nevertheless, sugar content and the other quality traits are known to demonstrate significant seasonal (Suthumchai et al., 2007) and year-to-year fluctuations (Mampholo et al., 2016), so further studies should assess G × E interactions and the optimal harvesting time for each landrace. Moreover information about the differences between landraces and modern varieties regarding other important quality traits such as nitrate content, carotenoid antioxidants and other compounds will be of great interest to boost the revaluation of these varieties.

Multivariate analyses, conducted on morphological descriptors (**Figure 2**) and chemical and color traits (**Figure 3**), revealed a consistent grouping for the Butterhead and Batavia accessions, and for the modern varieties of Oak leaf. By contrast, Oak leaf landraces were highly distinct to their modern counterparts, and Cos landraces showed a higher within-variety diversity. In the case of morphological descriptors, these included several traits not directly related to the external appearance of the mature lettuce (e.g., traits measured on seedling, young leaf, or stem), so these results can offer further clues regarding the phylogenetic relationships of each cultivar group. Cos lettuces have been described as one of the most ancient cultivated varieties (de Vries, 1997), and it has been hypothesized that the other cultivar groups have been derived from this source of variability (Mou, 2008). Our results show that Cos lettuces present a high intra-varietal diversity, which is in accordance with previous results obtained using molecular markers (Sharma et al., 2017).

Lettuce is a highly heterogeneous plant, which complicates methodological protocols to analyze quality traits. For instance, some correlations with chemical composition were significant solely when color or chlorophyll were measured in the terminal or equatorial part of the leaves (total sugars, chlorophyll, and color). Correlation between total sugars and chlorophyll content seem very interesting for breeders, as chlorophyll content has also been positively correlated with beta-carotene and lutein concentrations (Mou, 2005). Therefore, with farmers (and then breeders) initially selecting for green colored lettuces, they have in fact been selecting indirectly for increased sugar and carotenoid content. Nevertheless, the differences in composition between cultivar groups are very high (Mou, 2005; Simonne et al., 2001), and some correlations may be provoked by the differences between cultivar groups rather than because of pleiotropic effects. Thus, further research should focus on dissecting the genetic basis of these traits.

Considering that landraces are gaining interest in specific markets characterized by an emphasis on local production, organic farming and consumer demand for natural foods (Brush, 2000), we suggest that research programs intended to recover landraces should incorporate farmers and consumers in their phenotyping platforms. In our study farmers showed a high capacity to qualitatively characterize the genetic diversity related to the agronomic behavior. Moreover, consumers and farmers seem influenced by similar traits when scoring the varieties, being positively influenced by commercial weight (i.e., how intact the leaves of a variety appear), and negatively influenced by total weight and susceptibility to *B. lactucae*. Consumer agreement with farmer evaluations is particularly important, as it represents the potential to design an ideotype fulfilling the needs from both groups. Regarding lettuce color, it seems that consumers are particularly receptive to lettuces with intense red color on the internal part. By contrast, less interesting results were obtained when assessing the taste acceptance by consumers, probably due to the existence of different consumer segments, as reported for other crops (Causse et al., 2010), and their lower experience in characterizing materials.

## Conclusion

In agreement with previous analyses, this study identified the high intra-varietal diversity within the Cos cultivar group and characterized the principal differences with Butterhead, Batavia, and Oak leaf types. It showed that when comparing landraces with modern varieties, the principal factor of variance was related to the cultivar group. However, the higher scores for total sugars, dry matter, or chlorophyll content identified in landraces signals that these varieties show extremely promising characteristics. Regarding the agronomic behavior, yield, and resistance to the *B. lactucae* race isolated in the area were characterized in the germplasm collection, identifying one landrace that showed a high level of resistance. Finally, farmers showed a high technical capacity for characterizing the genetic diversity. It is therefore proposed that farmers and consumers should be included in the phenotyping platforms in future research projects aiming for the recovery of lettuce landraces.

## Author Contributions

JM made substantial contributions to the conception or design of the work; coordinated the field trials and phenotyping activities; participated in the analysis and interpretation of data; drafted the manuscript; and gave final approval of the version to be published and agreement to be accountable for all aspects of the work in ensuring that questions related to the accuracy or integrity of any part of the work are appropriately investigated and resolved. AR conducted the tasting sessions; participated in the analysis and interpretation of data; and revised the article critically, and final approval of the version to be published. BC made substantial contributions to the conception or design of the work; performed the agronomic characterization; organized the farmer evaluation; and interpreted the data. MF, CC, and SS made substantial contributions to the conception or design of the work; performed the laboratory tests for resistance to *B. lactucae*; and gave final approval of the version to be published. JS revised the article critically and gave final approval of the version to be published and agreement to be accountable for all aspects of the work in ensuring that questions related to the accuracy or integrity of any part of the work are appropriately investigated and resolved.

## Conflict of Interest Statement

The authors declare that the research was conducted in the absence of any commercial or financial relationships that could be construed as a potential conflict of interest.

## Footnotes

1 http://www.worldseed.org/our-work/plant-health/other-initiatives/ibeb/
